# Positive Impact of Late Harvest Date on Polyphenolic Composition of Plavac Mali (*Vitis vinifera* L.) Wine Depends on Location

**DOI:** 10.3390/foods13172695

**Published:** 2024-08-26

**Authors:** Ana Mucalo, Edi Maletić, Goran Zdunić

**Affiliations:** 1 Institute for Adriatic Crops and Karst Reclamation, Put Duilova 11, 21000 Split, Croatia; gzdunic@krs.hr; 2 Centre of Excellence for Biodiversity and Molecular Plant Breeding, Svetošimunska cesta 25, 10000 Zagreb, Croatia; emaletic@agr.hr; 3 Faculty of Agriculture, University of Zagreb, Svetošimunska cesta 25, 10000 Zagreb, Croatia

**Keywords:** anthocyanins, green berry wine, harvest date, flavan-3-ols, Mediterranean climate

## Abstract

Asynchronous ripening is a significant challenge in winemaking. Green berries reduce alcohol and pH while increasing acidity. Green berries are rich in bitter and astringent compounds, with an unknown impact on wine quality. The aim of this study was to evaluate the impact of harvest date and vineyard location on the polyphenolic composition of Plavac Mali wines in Dalmatia, Croatia. Experiments were conducted in two locations, Split and Zadar, producing fifteen wines per location from four harvest dates (H1–H4), including green berry wines from H1. The first harvest date occurred 27 days after véraison (DAV) and the last at 69 DAV, corresponding to overripeness. Green berry wines of H1 had low alcohol content up to 4.4% (v/v) in Split. Epigallocatechin was the main flavonoid in those wines, followed by dimer B1 in Split and catechin in Zadar. Green wines from Split had a higher concentration of phenolic acids, flavan-3-ol monomers and dimers. Wines of H3 had the highest concentration of malvidin-3-*O*-glucoside. With a later harvest date, a dramatic decrease in catechin and dimers was observed in wines from Split, and a decrease in epicatechin, epigallocatechin and dimer B1 in those from Zadar. The final expression of the physiochemical and polyphenolic composition of Plavac Mali wine is determined by the dynamics of harvest date, location and their interactions.

## 1. Introduction

The harvest date is a central premise in authentic wine production within a specific terroir, aiming to achieve phenolic and aromatic maturity for high-quality wines [1]. One of the main challenges in winemaking is asynchronous grape ripening, often managed by extending the harvest date [2]. Southern Mediterranean red wines, characterized by high alcohol and residual sugar content, achieve harmony by balancing prominent compounds such as alcohol and tannins. Iconic wines, often in the mid-alcohol category [3], can benefit from late harvests. Merlot harvested at 24.9 °Brix and pH 3.68 [4], and Shiraz at 25.5 °Brix and pH 4.03, exhibited improved sensory profiles and greater color intensity, anthocyanins, phenols, SO_2_-resistant pigments, and tannins compared to those harvested early [3].

However, late harvests can result in yield reduction [5], a 20% reduction in berry mass [6], shriveling, increased °Brix and pH due to dehydration, and susceptibility to *Botrytis cinerea*. The increase in anthocyanin concentration in wine, influenced by grape density [7], is attributed to higher concentrations in the skins. Structural changes in the cell walls due to pectolytic enzyme activity and enhanced extraction by hydroalcoholic solutions at higher ethanol concentrations further contribute to this effect. 

Grapes of early harvests are lower in total soluble solids (TSS) and higher in acidity and phenolic content, producing wines with more freshness, suitable for long-ageing [8]. Late harvest increases TSS and decreases extractable seed polyphenols, which are related to bitterness and astringency in wines, leading to more intense and varietal-like wines [9]. Late harvest dates result in a 48% increase in anthocyanins compared to ethanol adjustment, which has no effect on water-soluble anthocyanins [4].

In the Mediterranean climate, technological grape maturity is achieved faster than phenolic maturity [10]. Currently, well-structured red wines high in tannins, 14 to 16% v/v alcohol, and pH 4 are common due to climate change and viticulture techniques favoring high TSS [11]. These wines often lack acidity and optimal pH for long-term aging, with high alcohol contributing to perceived hotness.

Several techniques have been proposed to address these issues by increasing acidity and decreasing pH and alcohol through blending, such as early harvest, sequential harvest regimes, and the production of “green harvest wine” [7,12]. Early harvests can reduce alcohol and pH while increasing acidity. Kontoudakis et al. [7] suggested using odorless and colorless low-alcohol (~5% v/v) wines from grapes harvested at véraison for blending. This technique reduced the ethanol content of the final wine from 0.9 to 3.0% v/v, accompanied by an increase in total acidity from 0.8 to 2.2 g L^−1^. These changes vary depending on the specific characteristics of the wine matrix, derived from three different red varieties Cabernet Sauvignon, Merlot, and Bobal. 

However, the polyphenolic profile of green berry wines remains largely unexplored, with limited studies that have reported decreases in color intensity and total phenolic index in wines from early harvests [4,13]. Plavac Mali, a late-ripening red grape variety indigenous to the Mediterranean region of Croatia, produces premium, full-bodied wines with high phenolic content and strong antioxidant capacity [14]. This cultivar is prone to asynchronous ripening of berries within clusters, resulting in many green berries at later harvest dates [15,16], while these green berries can positively affect the wine’s acidity and pH and decrease the alcohol [7,12], their impact on wine quality and polyphenolic composition is still unclear. The proanthocyanidin composition of Plavac Mali berries significantly affects astringency and bitterness [7,12,17], with green berries being rich sources of these compounds [10]. 

The aim of this study was to extend previously published research by Mucalo et al. [10] on Plavac Mali grapes. This study examined the impact of four different harvest dates at two distinct agroecological subregions within Dalmatia, Split and Zadar, on the polyphenolic composition of Plavac Mali wines. Special focus is given to green berry wines produced on the first harvest date, as maintaining optimal grape acidity, pH, and lower sugar content at harvest is crucial from a climate change perspective. This research provides an in-depth analysis of the impact of four different harvest dates, two locations, and their interaction on the physiochemical parameters, flavonoid (anthocyanins, flavan-3-ols, and proanthocyanidins), nonflavonoid (phenolic acids) and stilbene compounds in Plavac Mali wines. This study gives comprehensive insight into the impact of harvest date and location on wine characteristics. 

## 2. Materials and Methods

### 2.1. Reagent, Solvent and Standard Chemicals

All chemicals used were of analytical grade for high-performance liquid chromatography (HPLC). Acetonitrile was purchased from J. T. Baker (Deventer, The Netherlands). Formic acid and orthophosphoric acid (85% w/w) were obtained from Fluka (Buchs, Switzerland) and ethanol from Kemika (Zagreb, Croatia). Milli-Q water was used for HPLC. Standard analytical grade phenolic compounds used for identification and quantification included delphinidin-3-*O*-glucoside, cyanidin-3-*O*-glucoside, peonidin-3-*O*-glucoside, malvidin-3-*O*-glucoside, epigallocatechin, procyanidin B1, procyanidin B2, rutin (quercetin-3-*O*-rutinoside), and myricetin from Extrasynthese (Lyon, France). Standards of (−)-epicatechin, (+)-catechin, (−)-epicatechin gallate, and quercetin-3-*O*-glucoside were obtained from Sigma-Aldrich (St. Louis, MO, USA), while kaempferol, quercetin, and isorhamnetin were sourced from Fluka (Steinheim, Germany).

### 2.2. Environmental Conditions and Vineyard Design

Experiments were conducted with cv. Plavac Mali cultivated in two germplasm repository vineyards located in different Dalmatian wine subregions. The first is located in Split–Duilovo (43°30′13.96″ N 16°29′56.467″ E, 14 m a.s.l.), within Central Dalmatia, while the second is situated in Zadar–Baštica (44°09′25.6″ N 15°26′12.6″ E, 120 m a.s.l.), within Northern Dalmatia. The climate in these areas is Mediterranean, with hot, dry summers and rainy winters. The 8-year-old vines in Split were grafted onto Boerner rootstock (*V. riparia* × *V. cinerea*) and trained to a spur-pruned bilateral cordon, with eight winter buds per vine. The vine spacing was 2.0 m × 1.0 m, resulting in a planting density of 5000 plants ha^−1^. The 5-year-old vines in Zadar were grafted onto the rootstock Kober 5BB (*V. berlandieri* × *V. riparia*) and trained to a unilateral spur-pruned cordon system. Vine spacing was 2.2 m × 1.1 m, and the planting density of 4545 plants ha^−1^. Both vineyards were located on brown soils on limestone, with north–south oriented rows, employing vertical shoot positioning and identical viticultural practices.

**Figure 1:**
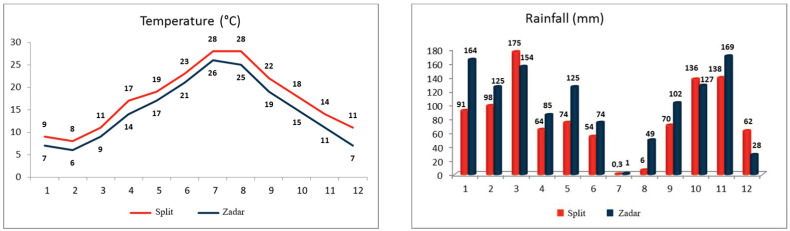
Monthly average temperature and rainfall for the two studied locations, Split and Zadar.

Although grape berry development and ripening can be influenced by the scion-rootstock combination [18], this study does not focus on these effects due to the differing terroir units of the vineyards. Previous studies reported the non-significant effects of Boerner and Kober 5BB rootstocks on polyphenolic biosynthesis compared to own-rooted vines [19]. Climatic conditions were monitored using meteorological stations near the locations (Figure 1).

Figure 1 illustrates the weather conditions, average monthly temperatures, and rainfall of the two studied vineyards, Split and Zadar. The data were obtained from the National Hydrometeorological Institute (DHMZ) meteorological stations Split-Marjan and Zadar-Zemunik, situated 3 km from the vineyards. The Split vineyard is situated in the warmest coastal region of the Adriatic, with average monthly temperatures that are 2 °C to 4 °C higher than those in Zadar. Additionally, the region experiences less precipitation, with the exception of March, October, and December. The grape harvesting and sampling occurred during the period of the highest average temperatures and the most extreme drought. The mean monthly temperature of the Split in August was 28 °C, with five days exceeding 30 °C. In contrast, the mean monthly temperature of Zadar in August was 25 °C, with a maximum temperature of 29 °C. The mean monthly temperature in September was 22 °C in Split and 19 °C in Zadar. The driest month at both locations was July. The total precipitation recorded at the Split in August was 6.2 mm, occurring after a period of 57 days with no rainfall. In August, the Zadar exhibited greater humidity, with 48.9 mm of precipitation occurring after 51 days of continuous aridity. The month of September marked the end of the extreme dry season, with a total precipitation of 70.3 mm and 102.2 mm, respectively.

### 2.3. Harvest Dates and Microvinification

Grapes were collected at four different harvest dates (H1–H4) from two vineyards, as previously described in [10]. The first harvest date (H1) was 27 days after véraison (DAV) when 90% of berries were colored; H2 was 41 DAV, H3, 55 DAV, and H4, 69 DAV, coinciding with overripeness. Harvest intervals were 14 days at each site, with specific dates being August 22 and 30 (H1), September 5 and 14 (H2), September 19 and 27 (H3), and October 3 and 12 (H4) for Split and Zadar, respectively. On each harvest date, 150 kg of grapes were manually harvested using a random and systematic sampling strategy to ensure sample representativeness at the vineyard level [20], with one vine harvested per interspace (comprising six plants). The total harvested mass of 150 kg of grapes per harvest date per location was divided into three lots, each weighing 50 kg. All green berries from the first harvest date were carefully separated by scissors from the whole grape amount. The green berries were weighed using a Kern 512 precision balance (Kern & Sohn GmbH, Balingen, Germany). A total of 8 kg and 9.3 kg of green berries were separated in Split and Zadar, respectively. These green berries were destemmed, manually crushed, and divided into three lots, each serving as a replicate. The fermentation of green berries pomace was conducted in 2 L Erlenmeyer flasks following the protocol used for colored berries. After mechanical destemming and crushing, sulfur dioxide (SO_2_) was added at a concentration of 35 mg L^−1^. Inoculation with the Lalvin EC1118 commercial yeast strain (Lallemand, Montreal, QC, Canada) followed at a concentration of 300 mg L^−1^. Alcoholic fermentation and maceration proceeded simultaneously for 6 days at 23 °C, with punch-downs performed twice daily. Upon completion of the primary aerobic phase of alcoholic fermentation, the pomaces were pressed and decanted into 20 L glass balloons and 1 L Erlenmeyer flasks. The anaerobic fermentation continued for three weeks. Following this period, the wines were decanted again and treated with 30 mg L^−1^ of SO_2_. The wines underwent a second decantation after an additional two months and were bottled into 0.75 L bottles.

### 2.4. Analysis of Flavonoid Compounds by HPLC

A high-performance liquid chromatography (HPLC) system equipped with diode array (DAD) and fluorescence detectors (FLD) (Agilent 1100, Palo Alto, CA, USA) was used for the identification and quantification of the targeted polyphenolic compounds in wines, following the method described by Tomaz and Maslov [21]. Prior to analysis, the wine samples were filtered using a 0.22 μm PTFE membrane filter (Milford, MA, USA) directly into the autosampler vial. Chromatographic separation was conducted on a Luna phenyl-hexyl column (Phenomenex, Torrance, CA, USA) (250 mm × 4.6 mm i.d., 5 μm particle size) with a phenyl guard column (4.0 × 3.0 mm), maintained at 50 °C. A 20 μL volume of the sample was injected into the HPLC system. Two mobile phases were employed as gradient solutions for elution: (A) water/phosphoric acid (99.5/0.5, v/v) and (B) acetonitrile/phosphoric acid/water (50/49.5/0.5, v/v/v). The flow rate of the mobile phase was 0.9 mL min^−1^. The detection of flavonoid compounds in the eluent via a nonpolar column of this reversed-phase chromatography system was performed using the DAD detector configured to an acquisition range of 200–700 nm. The detection of hydroxybenzoic acids was at 280 nm, hydroxycinnamic acid at 320 nm, flavonols at 360 nm, and anthocyanins at 518 nm using DAD. The flavan-3-ols were detected at an excitation wavelength (λex) of 225 nm and an emission wavelength (λem) of 320 nm by a fluorescence detector. The identification of analytes was accomplished by comparing the retention times of chromatographic peak with the external standard, DAD and fluorescence spectrum [21,22]. The quantitative values of the identified compounds were determined using a five single-point calibration curve, and the peak area of the corresponding standard compound was analyzed under identical conditions. The lowest regression coefficient of the calibration curve was 0.9957 for cyanidin-3-*O*-glucoside [21]. The results are expressed in mg L^−1^.

### 2.5. Analysis of Standard Components of Wine

Standard physicochemical oenological parameters of the wine were determined following the official methods of wine and must be analyzed [23]. These parameters are listed in Supplementary Table S1.

### 2.6. Statistical Analyses

A two-way analysis of variance (ANOVA) was conducted to evaluate the significance of the main effects of harvest date (Factor A) and locations (Factor B), as well as their interaction on polyphenolic and physiochemical parameters in wine samples. A total of 12 wine samples of four different harvest dates (H1 to H4) in three replications were analyzed per each of the two locations. Furthermore, to determine the differences among the four harvest dates within each location, one-way ANOVAs and mean separation using Fisher’s LSD test were conducted separately for each location. To determine the difference between green berry wines produced at H1 from each of the two locations, a one-way ANOVA and mean separation using Fisher’s LSD test were used. The statistical analyses were conducted using SAS software version 9.4 (SAS Institute, Inc., Cary, NC, USA). The results are presented as mean ± standard deviation. The reported uncertainties represent the standard deviations calculated from three replicates of each treatment.

## 3. Results and Discussion

### 3.1. Physiochemical Parameters and Phenolic Content in Green Berry Wines of H1 from Two Vineyard Locations

The green berry wines of H1 from two locations, Split and Zadar in Dalmatia, have a distinct physiochemical and phenolic composition. Green wines are characterized by low alcohol content, low pH and high total acidity, which makes them interesting for enhancing wine stability compared to wines of colored berries from H1. Additionally, volatile acid, ash and total extract content would not impair the final quality of the wine if added in order to reduce overall alcohol content. What intrigues us more is the phenolic composition of green berry wine, as shown in Table 1. 

The green berry wines of the two locations significantly differ in the concentration of quercetin-3-*O*-glucoside, caffeic, fertaric, and gallic acid, and gallic esters of catechin, epicatechin gallate and epigallocatechin. In the wines from Split, each of these compounds was present in a higher concentration than in wines from Zadar. The most abundant compound in both green berry wines was epigallocatechin, with concentrations of 124.76 mg L^−1^ and 67.67 mg L^−1^ in Split and Zadar, respectively. In wines from Split, it was followed by dimer B1, and in wines from Zadar by catechin. 

### 3.2. Evaluation of the Standard Composition of the Wines at Four Different Harvest Dates from Two Vineyard Locations

The evaluation of the standard physicochemical parameters of the wines produced from colored berries of H1 to H4 in both locations, Split and Zadar, is shown in (Table S1). The typical patterns of a gradual increase in ethanol concentration, from 9.9 ± 0.1% (v/v) to 14.4 ± 0.6% (v/v), and a decrease in total acidity from 6.4 ± 0.1 g L^−1^ to 5.9 ± 0.0 g L^−1^, from H1 to H4 are seen in the wines from Split. In the case of Zadar, similar patterns of those compounds are seen until H3, with a decrease in ethanol and an increase in total acidity afterward in H4 in comparison to wines from Split. Moreover, in Zadar, these changes are accompanied by a decrease in residual sugar and an increase in volatile acidity. Wines of prolonged ripening (H4) from the vineyard located in Split had a significantly higher content of total dry extract (42.4 ± 8.4 g L^−1^), residual sugar (14.1 ± 8.7 g L^−1^), ash (3.5 ± 0.1 g L^−1^), pH (3.7 ± 0.0) and volatile acidity (0.5 ± 0.0 g L^−1^). In the case of Zadar, the highest pH of 3.4 and lowest total acidity of 6.4 g L^−1^ were in wines of H3. 

### 3.3. Flavonoid Indicators of Grape Ripeness in Wines of Four Harvest Dates from Two Vineyard Locations

**Figure 2:**
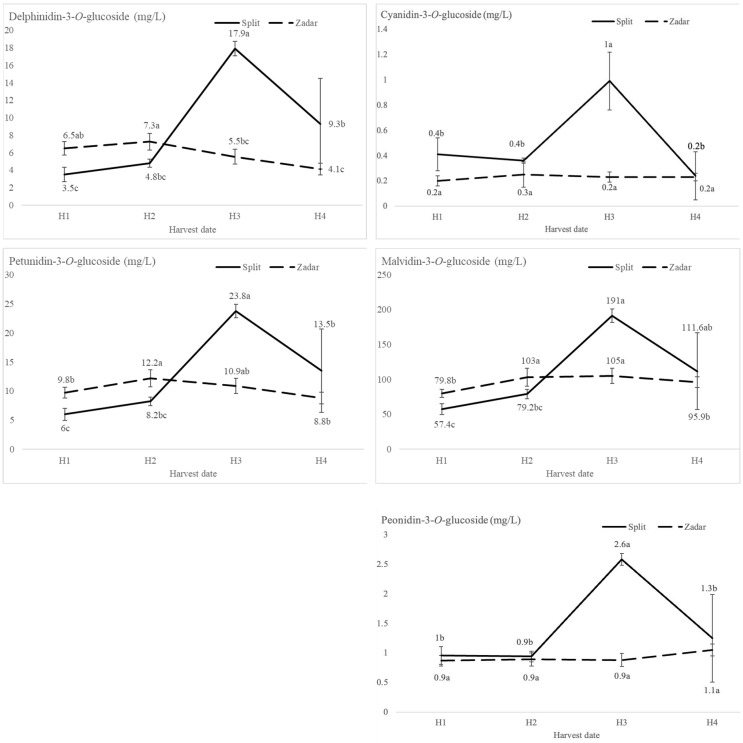
Anthocyanin compositional analysis of Plavac Mali wines of different harvest dates where H1 was the earliest, and H4 was the latest harvest date from two locations (Split and Zadar) determined by HPLC-DAD. Values with different letter (a, b, c) are significantly different, according to the Fisher’s LSD test (*p* < 0.05).

The harvest date significantly affected the patterns of phenolic compounds in the wine. The wines of H3 from Split reached peak values of anthocyanidin-3-*O*-monoglucosides, followed by a significant decrease of each except malvidin-3-*O*-glucoside, as shown in Figure 2. On the contrary, the breakpoint for anhocyanidin-3-*O*-glucoside patterns in the Zadar wines occurred at H2. After this point, the most dominant anthocyanin, malvidin-3-*O*-glucoside [14], reached its peak value in H3 (104.95 mg L^−1^), and peonidin-3-*O*-glucoside in H4 (1.05 mg L^−1^). 

Wines of early harvest dates had the highest concentration of quercetin glycoside, regardless of location, as shown in Table 2. In the wines of the H4, quercetin aglycone dominates only in Split. On the contrary, in Zadar wines, the decrease in quercetin-3-*O*-glucoside with later harvests was not accompanied by an increase in free quercetin fraction.

The wines of early harvest dates (H1 and H2) from both locations had significantly higher concentrations of catechin than those from H3 and H4. As the harvest was postponed, the catechin trends followed different patterns at the two locations. In Split, the concentration decreased to 15.23 mg L^−1^ in H4, while in Zadar, it remained stable during H3 and H4 and reached 30.22 mg L^−1^ in H4. The concentration of epicatechin gradually decreased, reaching a minimum at H3 in Split, while an opposite trend was seen in Zadar wines.

In Plavac Mali wines of each harvest date, epigallocatechin is the most abundant flavan-3-ol monomer. The wines of H2 from Split exhibited significantly lower concentrations of epigallocatechin. In wines from Zadar, a dramatic decrease in epigallocatechin is seen in H4. 

A decrease in procyanidin dimers occurred from H2 in wines from both locations. In wines of early harvest dates from Split, dimer B2 dominates, while in wines of latter harvest dates, the most dominant one was B1. In wines from Zadar, procyanidin B4 followed B2. The quantitative and compositional differences in procyanidins extracted from seeds based on harvest date prolongation appear to vary depending on the vineyard location. Specifically, the initial concentration of anthocyanins and seed proanthocyanidins in each location could affect the formation of polymeric pigments differently. 

### 3.4. Nonflavonoid Indicators of Grape Ripeness in Wine of Four Harvest Dates from Two Vineyard Locations

The content of hydroxycinnamoyl tartaric acids, caftaric, coutaric, and fertaric, showed a sharp decrease with a delay in harvest date regardless of location, as shown in Table 3. Wines of H1 had the highest concentrations of caftaric and its precursor caffeic acid, as well as coutaric and its precursor coumaric acid, regardless of location. The opposite trend, of an increase in fertaric acid in wines of later harvest dates, was observed at both locations, even though its precursor ferulic acid was not detected in wines from Zadar. The peak of hydroxybenzoic gallic acid was measured in the wines from H2 of both sites. In the wines of later harvest date from Split, a sharp decrease in gallic acid occurred. Conversely, wines from Zadar showed a variable decrease in gallic acid in later harvest dates. 

The wines of H3 and H4 in Split had significantly higher concentrations of resveratrol than the wines of previous harvest dates. In the wines of Zadar, the maximum concentrations were in H2, with a significant decrease afterward. The concentration of resveratrol glucoside in wines of Split and Zadar increased progressively across the four harvest dates, with the highest values observed in H4 at both locations. 

### 3.5. Discussion on Physiochemical Parameters and Phenolic Composition of Wines of Four Different Harvest Dates from Two Locations

The differences in the phenolic composition of green wines of H1 from both locations indicate that specific local agroecological conditions influence the transition of green berries from growth to the lag phase [24] and change the expression level of phenylpropanoid pathway genes. Previously, the earlier up-regulation of VviPAL expression in green Agiorgitiko berries in response to drought was reported [25]. The Split region experienced warmer and drier conditions, with temperatures reaching a maximum of 28 °C in August and a 57-day period of summer drought. In contrast, the Zadar vineyard exhibited cooler temperatures, with a maximum of 25 °C in August and higher humidity, but also a 51-day period of drought. In particular, berries from Split had lower total mass and seed mass but a higher total number of seeds in comparison to those from Zadar [10]. An increase in seed number has been linked to hormonal balance and maturation of berries, affecting anthocyanin content [26]. The onset of the ripening phase is observed 4 to 14 days earlier in berries with lower seed content than in those with higher seed content. Moreover, those with a higher seed weight compared to those with a berry weight typically remain green with low sugar content [27]. The unique green wine matrix composition favored the extraction of phenols from unripe seeds over skins, particularly evident in wines from Split, which exhibited a higher content of gallic acid and gallate-type catechins. This, together with a higher concentration of quercetin-3-*O*-glucoside, aligns with the enhanced extraction capability of low-alcohol mixtures [28]. The differential extraction is attributed to varying polarities and compound-specific responses mediated by matrix differences [29]. While green wines are a rich source of acids, contributing to bitterness and astringency, this research suggests caution regarding their potential, as these can seriously impair the quality of future wine. 

The physiochemical composition of wines produced from colored berries of H1 to H4 in Split of an increase in ethanol, and a pH and a decrease in total acidity are in accordance with results reported for Cabernet Sauvignon [7,20]. A decrease in ethanol, reducing sugars, and an increase in total and volatile acidity in wines of H3 to H4 from Zadar could be due to heterogeneity of berries [7]. The high residual sugar content in H4 wines from Split indicates stuck alcoholic fermentation, which is consistent with the lower yeast fermentation efficiency at high pH in H4 and the need for acid adjustment [20]. In general, low pH and high total acidity in wines from Zadar are positive aspects of vineyard relocation to the northern location, as these are important for maintaining the microbiological and physicochemical stability, as well as the freshness of wines [30]. The harvest date significantly affected the patterns of phenolic compounds in the wine [13]. The wines of H3 from both locations exhibited peak values of malvidin-3-*O*-glucoside. Previously, the highest concentration of malvidin-3-*O*-glucoside in the skin extract of Plavac Mali berries was also reported in H3, irrespective of location [10]. The peak concentrations of anthocyanins in wine are linked to phenolic maturity, the concentration of anthocyanins in the skin, and the ease of their extraction into the wine [31,32]. The discrepancy between quercetin glycoside and free quercetin fraction, a product of acid hydrolysis [33] in late-harvest wines from Zadar, may be due to differences in the solubility of quercetin aglycone, which is proportional to ethanol concentration in the solution [34]. Additionally, quercetin’s strong binding ability, as a main copigment, could also contribute to this variation [29,35]. 

The decrease in catechin concentrations with later harvest dates in Split is consistent with those reported for Tinto Fino and Cabernet Sauvignon [36]. However, stable concentrations in wines of H3 and H4 in the case of Zadar could be indicators of lower ripeness of these berries. Catechin and epicatechin are the most abundant flavan-3-ol monomers in grape seed extracts of Plavac Mali, with minimal concentrations of each in H3 and H4 of both locations [10]. The minimal ratio between these two isomers in wines of later harvest dates, H4 in Split and H3 and H4 in Zadar indicate tannin oxidation. 

In wines of later harvest dates (H3 and H4) from Split, a higher ratio of trihydroxylated gallocatechins, which have a lower affinity for salvia proteins compared to dihydroxylated catechins, is seen. Gallocatechins are associated with smothering, more velvety, and viscous sensations in wine [37]. The dominance of epigallocatechin in wines of Plavac Mali, regardless of harvest date, is associated with the lower bitter intensity of these wines [17]. The predominance of epigallocatechin in Plavac Mali wine is a grape skin extraction indicator, as it is one of the main building blocks of prodelphinidins [38,39]. Increased extraction of skin prodelphinidins, while seed proanthocyanidins remain relatively constant, is desirable from a winemaking standpoint. 

One of the indicators of the relative proportion of proanthocyanidin extraction from the skin and seed into wine is the ratio between epigallocatechin and epicatechin [40]. The highest ratio of these two wines from Split is in H3 due to the lowest extractability of proanthocyanidins from seeds at the time of phenolic maturity [32,41]. Enhanced extraction of skin over highly galloylated seed-derived phenolics [40,42] with an extension of harvest date is consistent with findings on Cabernet Sauvignon [13]. On the contrary, in wines from Zadar, the maximum was in H1, in accordance with the predominance of skin proanthocyanidins in early harvest dates and seed proanthocyanidins in later harvest dates with a higher ethanol content.

In wines from the late harvest dates H3 and H4, a decrease in procyanidin dimers was observed across both locations. In Split, procyanidin dimer B1 was dominant, while in Zadar, dimer B2 was more prevalent. Previously, a dominance of dimer B1 at the grape skin and seed level in Plavac Mali was reported. In the grape seed extracts, regardless of the harvest date at both locations, B1 was followed by dimer B2 [10]. Interestingly, when Casassa et al. [4] compared the proportion of skin and seed-derived proanthocyanidins in Merlot wines as a function of grape ripeness, over 70% of them were seed origin in the two-year study. The extraction of proanthocyanidins from grape seeds reached its peak at an ethanol threshold of 11.7% v/v. Beyond this threshold, further increases in ethanol concentration did not necessarily lead to higher extraction of proanthocyanidins. The quantitative and compositional differences in procyanidins extracted from seeds based on harvest date prolongation appear to vary depending on the vineyard location. Specifically, the initial concentration of anthocyanins and seed proanthocyanidins in each location could affect the formation of polymeric pigments differently. The higher temperatures and lower precipitation in Split could be associated with higher concentrations of anthocyanins [43] but also face a risk of degradation of those compounds during extreme heat [44]. The cultivation of Malbec and Bonarda under temperatures exceeding 40 °C showed a reduction of total anthocyanins by up to 41% and an increased proportion of acylated anthocyanins in comparison to the control treatment [45].

The high concentrations of anthocyanins and proanthocyanidins enhance the formation of polymeric pigments in wine [46]. This highlights the complex interplay of grape chemistry and environmental factors in determining wine composition and quality. In addition to differences in extraction kinetics, flavonoids exhibit varying degrees of stability and binding capacity. Quercetin-3-*O*-glucoside has a strong binding capacity, followed by epicatechin and gallic acid [29,35]. This is dependent on the hydroxylation and methoxylation pattern on the B ring, *O*-glycosylation, and acylation [29]. The electron-donating capacity of molecules affects their ability to engage in copigmentation reactions [47]. This strongly affects how these molecules interact within the wine matrix, influencing their stability and contribution to wine color and flavor. 

Moreover, the composition of the wine matrix itself (ethanol, acids, pH, sugars, and proteins) significantly impacts the phenolic composition of wines. Factors such as pH and ethanol content have a strong impact on the stability of anthocyanins. Higher pH levels tend to favor hydration reactions, which can alter the color and stability of wine pigments. Ethanol, through its dissociation effect, also impacts copigmentation complexes, further influencing the phenolic profile and color stability of the wine [48]. Therefore, understanding these complex interactions between flavonoid properties, wine matrix composition, and environmental factors is crucial for managing and enhancing wine quality, color stability, and flavor profile during winemaking and aging processes. 

The declining trend in phenolic acids in wines of later harvest dates observed in both locations aligns with previous findings indicating a decrease during later ripening stages [49]. This may be attributed in part to the formation of more stable and soluble anthocyanin-derived pigments incorporating caffeic or *p*-coumaric acid [50,51]. Additionally, a significant decrease in the concentration of anthocyanin-3-*O*-monoglucosides between H3 and H4 wines from Split support this trend, consistent with an increase in malvidin-3-(6”-*O*-coumaryl) glucoside pigment in Plavac Mali skins with prolonged ripening [52]. During alcoholic fermentation, phenolic acids undergo various rapid processes, such as oxidation or slower processes, such as hydrolysis and *cis*-isomerization. 

Gallic acid is extracted from the seeds [33], which are typically found in ester-bound forms. In the wines of later harvest dates from Split, a sharp decrease in gallic acid is in accordance with the ripening associated decrease and desiccation of grape seeds [41,49]. Conversely, wines from Zadar showed a variable decrease of gallic acid in later harvest dates, and this can be attributed to enhanced extraction efficiency from unripe seeds due to physiochemical characteristics of the wine, including increased ethanol content and lower pH value [4,53]. 

Increases in resveratrol and resveratrol glucoside in wines of H3 and H4 from Split could be associated with high ultraviolet light exposure [54]. In contrast, high average temperatures of 42 and 35 °C, in comparison to maximum daily temperatures of 26 and 21 °C, inhibited the synthesis of these compounds in Sangiovese grapes [55]. A decrease in resveratrol and an increase in resveratrol glucoside with later harvest dates in wines from Zadar could be due to the glycosylation of resveratrol, influenced by grape sugar content. In conditions in which grapes accumulate more sugars, the formation of glucosides may be favored, resulting in a higher glucoside-to-resveratrol ratio [56].

### 3.6. Effect of Location on Physiochemical and Phenolic Composition of Wines

In order to gain insight into the impact of location on wine composition, a two-way analysis of variance was performed on the physiochemical and phenolic parameters of the wines. Significant differences were found at various confidence levels for different parameters, with *p* ≤ 0.05 for reducing sugars and *p* ≤ 0.01 for total dry extract. Significant differences at *p* ≤ 0.001 were found for ethanol, ash, pH, total acidity, and volatile acidity. The wines from Split had higher concentrations of each of these parameters, except for total acidity, in comparison to wines from Zadar. These differences can be attributed to the varying agroecological conditions of the two locations, with average monthly temperatures 2 °C to 4 °C higher in Split compared to Zadar. The most important factors affecting vegetative growth, yield formation, and berry composition at harvest are soil moisture and weather conditions during the growing season [57]. Late-ripening varieties require a certain amount of cumulative temperature and sun exposure to ensure the complete ripening of berries. High temperatures are associated with smaller berries, the promotion of TSS accumulation, and the thermal degradation of malic acid, leading to advanced technological maturity. Under extreme temperatures, photosynthesis and photosynthate translocation are reduced, resulting in uneven ripening, decreased TSS accumulation, and delayed ripening [58]. The agroecological conditions at Split, characterized by higher temperatures and potentially lower soil moisture, likely contributed to the observed differences in wine composition when compared to Zadar.

Significant differences at the *p* ≤ 0.05 were observed for fertaric acid, catechin, delphinidin-3-*O*-glucoside, and petunidin-3-*O*-glucoside. The concentration of each of these compounds was approximately twice that observed in wines from Zadar, except for catechin, which was present at half the concentration. Previous studies on Plavac Mali have reported different results on grape skin and seed extracts. Significantly lower concentrations of malvidin-3-*O*-glucoside and higher catechin were observed in the skin extracts from Split. In contrast, catechin in seed extracts from Split was found to be present at significantly lower concentrations compared to those from Zadar [10].

The wines from the two locations showed significant differences at the *p* ≤ 0.01 for peonidin-3-*O*-glucoside and highly significant differences at *p* ≤ 0.001 for cyanidin-3-*O*-glucoside, flavonols (myricetin-3-*O*-glucoside, quercetin-3-*O*-glucoside, quercetin and isorhamnetin), all flavan-3-ols (except catechin), proanthocyanidins, and stilbenes. The wines from Split had a higher concentration of each of these compounds, except epicatechin and proanthocyanidins B2 and B4. These compounds may serve as markers to distinguish wines based on their vineyard of origin. Previously, the perfect differentiation of wines following bottle aging from the regions of Côte de Beaune and Côte de Nuits, situated 30 km apart, was achieved based on their terroir chemical fingerprint. Moreover, the effect of location on the phenolic composition overcame the effect of seasonal variations. This was not observed in the case of grape skin extracts, must analyses or wines immediately after alcoholic fermentation [59]. This suggests that the location-specific differences in phenolic composition become more pronounced with aging, highlighting the importance of terroir in the final wine profile. The anthocyanin composition is used as a chemotaxonomic marker for the classification of wines based on their varietal and geographical origin [60]. Almost the same concentrations of the main malvidin-3-*O*-glucoside and generally low concentrations of peonidin-3-*O*-glucoside and cyanidin-3-*O*-glucoside at both locations serve to confirm the grape variety. The two experimental locations differ mostly in climate conditions and vigor-related components. The Split is classified as a Winkler V (hot) region based on the sum of effective temperatures during the vegetation cycle, while Zadar is classified as a Winkler IV (moderately warm) region [61]. 

The low vegetative area of the training system under drought conditions in Split is associated with higher solar radiation in the fruiting zone, which promotes the synthesis of flavonols [62]. This is supported by the observed decrease in anthocyanin-3-*O*-monoglucosides with prolonged ripening can be attributed to the thermal degradation of these compounds at high temperatures [44] or to a compositional shift towards malvidin-based anthocyanins and a higher rate of acylation during biosynthesis, which increases the stability of anthocyanins [62]. Compositional shifts in the condensation of proanthocyanidins from seed and skin in response to changes in light exposure have been reported [63]. These shifts have been observed to result in a catechin content that is proportionally higher than epicatechin in berries from low-vigor vines. Wines from Zadar have higher total acidity, lower pH, and higher seed procyanidin content, which can be associated with a greater day/night thermal amplitude [64], maximum temperatures below 30 °C, lower UV irradiation during ripening, and reduced desiccation of grape seeds under humid conditions [65]. The production of fully colored wines with a high concentration of anthocyanins, flavonols, and phenolic acids was obtained in Split at H3, with an alcohol content of 12.8% v/v. The wines obtained with prolonged ripening (H4) had an alcohol content of 14.4% v/v and a slightly lower concentration of each of these compounds. This is consistent with previous findings on Cabernet Sauvignon and Shiraz, which indicate that anthocyanin extraction is more dependent on the stage of maturation than on the alcohol content of the medium [53]. Although grape skin extracts from the Zadar had significantly higher concentrations of anthocyanins [10], the wines produced demonstrated consistently lower concentrations of these compounds at each harvest date.

Additionally, there was a predominance of bitter flavan-3-ol monomers, epicatechin and catechin, along with B2 and B4 dimers in wines from Zadar. This could indicate a higher extractability of these compounds from seeds. Despite the advantages of higher total acid content, lower pH, and lower alcohol values, wines from Zadar have lower qualitative potential based on their polyphenolic composition. This is likely due to the fact that the berries did not reach phenolic ripeness.

### 3.7. Impact of the Interaction Effects of ‘Harvest Date and Location’ on Physiochemical and Phenolic Composition of Wines

Highly significant two-way interaction effects of harvest date and location factors were observed at *p* ≤ 0.01 for total dry extract and residual sugar and at *p* ≤ 0.001 for the rest of physiochemical parameters (ethanol, ash, pH, total acidity and volatile acidity). The analysis showed that the interaction effect of harvest date and location was not statistically significant for epicatechin gallate, catechin, and B3. The observed trends for these compounds were consistent across different harvest dates despite the clear agroecological differences between the two vineyard locations.

Highly significant two-way interaction effects at *p* ≤ 0.01 for B1, B4, coumaric acid and resveratrol glucoside, and at *p* ≤ 0.001 for the rest of the phenolic compounds. The significant interaction indicates that the influence of the location factor on the compound of interest is not constant and varies significantly as a function of the harvest date (H1 to H4). Conversely, the influence of the harvest date on the compounds varies significantly between the different locations. This indicates that the effect of one factor is dependent on the level of the other factor, demonstrating the complexity of the interaction between agroecological conditions and harvest date on the composition of wines.

## 4. Conclusions

The final expression of the Plavac Mali variety in wine is determined by four harvest dates, the two geographical locations, and the dynamic interaction between these factors. The green berry wines from the Dalmatian vineyards of Split and Zadar show distinct physiochemical and phenolic profiles, characterized by low alcohol content, low pH, high total acid content, and epigallocatehin as the predominant flavonoid. This study highlights the complex interplay between harvest dates and location on the phenolic profiles of wines. The harvest dates significantly impact phenolic patterns observed in both locations. The early harvests (H1 and H2) have higher concentrations of catechin, proanthocyanidins, phenolic acids (with the exception of fertaric and ferulic acid), and resveratrol glucoside, which decrease in the later harvests (H3 and H4) in wines from both locations. Later harvests in Split increased the concentration of malvidin-3-*O*-glucoside, isorhamnetin, gallocatechin, epigallocatechin in H3 and H4, and rutin and quercetin in H4 wines. The Zadar wine shows increased concentrations of myricetin-3-*O*-glucoside and epicatechin in H3. 

The Split wines have higher concentrations of phenolic compounds, including anthocyanins, flavonols, flavan-3-ols (except gallocatechin in H1, and catechin and epicatechin), and proanthocyanidin dimers (except B2 and B4). Additionally, these wines contain higher concentrations of non-flavonoid phenolic acids, with the exception of fertaric and gallic acids, in later harvest dates. The phenolic ripeness of a grape variety is highly dependent on the terroir in which it is cultivated. The lack of phenolic ripeness observed in higher latitude vineyards of Zadar indicates a low polyphenolic plasticity of Plavac Mali. The modulation of the wine polyphenolic profile through the selection of harvest dates at each location allows for enhanced differentiation of wines in the market. By understanding terroir-specific phenolic ripeness, winemakers can adjust viticultural techniques to synchronize technological and phenolic ripeness, emphasizing the necessity for climate adaptation measures in vineyard management. The study offers valuable insights for winemakers, contributing to a deeper understanding and adaptation of viticultural practices to changing climatic conditions and effectively producing wines that reflect the unique terroir of each subregion.

## Figures and Tables

**Table 1:** One-way ANOVA showing mean separation of basic physiochemical and phenolic composition of Plavac Mali wines of green berries separated at H1 from two locations, Split and Zadar.

Parameter Group	Parameter	Split	Zadar
Basic physicochemical	Ethanol (% v/v)	4.40 ± 0.10 a	3.97 ± 0.12 b
	Residual sugars (g L^−1^)	4.28 ± 0.54 a	3.12 ± 1.30 a
	pH	2.90 ± 0.01 a	2.86 ± 0.01 b
	TA (g tartaric acid L^−1^)	12.47 ± 0.06 b	15.53 ± 0.06 a
	Acetic acid (g L^−1^)	0.23 ± 0.01 a	0.19 ± 0.01 b
	Ash (g L^−1^)	2.21 ± 0.07 a	1.83 ± 0.05 b
	Total dry extract (g L^−1^)	33.33 ± 0.55 a	31.33 ± 0.29 b
Flavonols	Q-3-*O*-g	22.69 ± 4.31 a	10.83 ± 1.23 b
Phenolic acid	Cft	55.76 ± 9.34 a	49.52 ± 1.55 a
	CA	10.10 ± 0.34 a	6.83 ± 0.15 b
	CtA	20.28 ± 5.03 a	15.81 ± 0.44 a
	CmA	3.28 ± 0.41 a	2.82 ± 0.08 a
	FtA	1.07 ± 0.16 a	0.56 ± 0.03 b
	FA	0.63 ± 0.15 a	0.48 ± 0.03 a
	GA	25.99 ± 3.60 a	11.83 ± 0.82 b
Flavan-3-ol monomers	ECG	3.12 ± 0.82 a	0.33 ± 0.04 b
	GC	6.76 ± 2.83 a	5.34 ± 1.42 a
	EGC	124.76 ± 22.99 a	67.67 ± 7.25 b
	C	69.12 ± 21.70 a	57.19 ± 2.03 a
	EC	18.06 ± 7.43 a	10.54 ± 1.68 a
Dimers	B1	87.35 ± 27.14 a	43.93 ± 5.09 a
	B2	23.12 ± 10.41 a	11.76 ± 1.82 a
	B4	29.22 ± 5.34 a	26.80 ± 1.24 a

Values are means with standard deviations of three separate repetitions. Values with different letter (a, b) in the same row are significantly different, according to the Fisher’s LSD test (*p* < 0.05). TA—total acidity (expressed in g L^−1^ as tartaric acid), Q-3-*O*-g—quercetin-3-*O*-glucoside, Cft—caftaric acid, CA—caffeic acid, CtA—coutaric acid, CmA—coumaric acid, FtA—fertaric acid, FA—ferulic acid, GA—galic acid, ECG—epicatechin gallate, GC—gallocatechin, EGC—epigallocatechin, C—catechin, EC—epicatechin, B1—procyanidin B1, B2—procyanidin B2, B4—procyanidin B4.

**Table 2:** Two-way and one-way ANOVA analysis of flavonoid composition of Plavac Mali wines of four different harvest dates from two locations, Split and Zadar, and the interactive effect of both factors.

Phenolic Group		Split				Zadar				L	HD × L
		H1	H2	H3	H4	H1	H2	H3	H4		
Flavonols	M-3-*O*-g	2.93 ± 0.18 b	4.45 ± 0.15 a	4.24 ± 0.15 a	2.32 ± 0.35 c	1.28 ± 0.02 B	1.23 ± 0.25 B	1.62 ± 0.18 A	1.37 ± 0.02 AB	***	***
	Rt	nd	nd	nd	0.82 ± 0.03	nd	nd	nd	nd	ns	ns
	Hy	0.70 ± 0.02 b	0.78 ± 0.04 a	0.61 ± 0.03 c	nd	nd	nd	nd	nd	ns	ns
	Q-3-*O*-g	15.54 ± 0.62 a	15.71 ± 0.76 a	12.79 ± 0.43 b	9.93 ± 0.07 c	2.60 ± 0.18 A	1.42 ± 0.24 B	0.69 ± 0.02 C	0.13 ± 0.04 D	***	***
	Q	4.91 ± 0.21 c	6.39 ± 0.22 b	7.31 ± 0.48 b	9.09 ± 0.92 a	2.05 ± 0.27 A	2.61 ± 0.67 A	2.34 ± 0.25 A	1.20 ± 0.16 B	***	***
	Kem	0.66 ± 0.03 a	0.67 ± 0.02 a	0.70 ± 0.08 a	0.72 ± 0.14 a	nd	nd	nd	nd	ns	ns
	Iso	0.55 ± 0.02 d	0.74 ± 0.02 c	1.01 ± 0.05 b	1.16 ± 0.09 a	0.42 ± 0.06 B	0.54 ± 0.06 A	0.53 ± 0.04 A	0	***	***
Flavan-3-ol monomers	ECG	5.17 ± 0.72 a	6.01 ± 0.14 a	5.61 ± 0.18 a	6.03 ± 1.36 a	1.80 ± 0.06 B	2.74 ± 0.47 A	2.85 ± 0.49 A	2.44 ± 0.48 AB	***	ns
	GC	1.03 ± 0.26 d	1.43 ± 0.08 c	1.79 ± 0.03 b	2.57 ± 0.10 a	1.63 ± 0.41 A	0.63 ± 0.21 C	0.85 ± 0.13 BC	1.18 ± 0.07 AB	***	***
	EGC	106.66 ±2.62 a	96.28 ± 0.80 b	104.48 ±1.92 a	108.11 ±5.54 a	73.80 ± 2.04 A	68.13 ± 1.44 B	55.40 ± 1.39 C	44.36 ± 2.03 D	***	***
	C	40.41 ± 0.89 a	31.90 ± 0.75 b	21.77 ± 0.95 c	15.23 ± 1.74 d	62.05 ± 0.91 A	44.54 ± 2.37 B	33.17 ± 1.23 C	30.22 ± 2.22 C	*	ns
	EC	5.91 ± 0.44 a	5.04 ± 0.47 b	3.00 ± 0.16 c	3.48 ± 0.23 c	8.89 ± 1.41 C	10.59 ± 0.86 B	12.45 ± 0.56 A	10.79 ± 0.10 B	***	***
Proanthocyanidins	B1	19.20 ± 0.55 a	13.50 ± 0.40 b	12.37 ± 0.90 b	9.77 ± 1.30 c	12.97 ± 0.87 A	10.00 ± 0.28 B	8.17 ± 0.28 C	5.78 ± 0.28 D	***	*
	B2	22.46 ± 0.90 a	17.32 ± 0.45 b	11.0 ± 0.57 c	6.28 ± 0.55 d	43.41 ± 0.45 A	31.40 ± 1.89 B	22.65 ± 1.09 C	21.85 ± 2.10 C	***	***
	B3	5.12 ± 0.23 a	3.77 ± 0.30 b	3.30 ± 0.38 b	2.47 ± 0.56 c	3.83 ± 0.66 A	2.82 ± 0.33 B	1.76 ± 0.13 C	1.33 ± 0.25 C	***	ns
	B4	12.28 ± 0.69 a	9.57 ± 0.24 b	6.60 ± 0.64 c	3.86 ± 0.50 d	15.24 ± 0.40 A	11.77 ± 1.04 B	8.00 ± 0.42 C	8.10 ± 0.10 C	***	*

Two-way ANOVA shows the mean separation of phenolic groups (PG): the flavonoid components (flavonol, flavan-3-ol, and proanthocyanidins) of Plavac Mali wines produced from berries harvested at four different dates (H1, H2, H3, and H4) at two locations (L) (Split and Zadar), and interactive effect (HD × L) of these factors (ns—not significant; * *p* ≤ 0.05; *** *p* ≤ 0.001). One-way ANOVA was done separately for each vineyard location. Mean values ± standard deviation (*n* = 3) within the same line, followed by different lowercase and uppercase Latin letters, indicate significant differences according to Fisher’s LSD test at *p* ≤ 0.05 among different harvest dates for Split and Zadar, respectively. M-3-O-g—myricetin-3-O-glucoside, Rt—rutin, nd – not detected, Hy—hyperoxide, Q-3-O-g—quercetin-3-O-glucoside, Q—quercetin, Kem—kaempferol, Iso—isorhamnetin, ECG—epicatechin gallat, GC—gallocatechin, EGC—epigallocatechin, C—catechin, EC—epicatechin, B1—procyanidin B1, B2—procyanidin B2, B3—procyanidin B3, B4—procyanidin B4. Values are presented in units of mg L^−1^.

**Table 3:** Two-way and one-way ANOVA analysis of non-flavonoid composition of Plavac Mali wines of four different harvest dates from two locations, Split and Zadar, and the interactive effect of both factors.

Compounds			Split				Zadar					
			H1	H2	H3	H4	H1	H2	H3	H4	L	HD × L
Phenolic acids		CfT	41.63 ± 2.30 a	35.01 ± 1.05 b	24.36 ± 0.47 c	20.67 ± 0.79 d	19.17 ± 0.79 A	15.70 ± 1.07B	13.14 ± 0.09C	8.69 ± 0.49 D	***	***
		CA	6.09 ± 0.30 a	5.62 ± 0.14 b	4.63 ± 0.09 c	3.70 ± 0.11 d	2.83 ± 0.07 A	2.56 ± 0.17 B	2.36 ± 0.04 C	1.88 ± 0.10 D	***	***
		CtA	13.63 ± 0.94 a	11.96 ± 0.32 b	9.26 ± 0.19 c	7.46 ± 0.24 d	6.27 ± 0.16 A	4.85 ± 0.38 B	4.38 ± 0.09 C	3.10 ± 0.16 D	***	***
		CmA	2.78 ± 0.13 a	2.57 ± 0.02 b	2.48 ± 0.02 bc	2.37 ± 0.01 c	1.76 ± 0.03 A	1.66 ± 0.12 A	1.65 ± 0.01 A	1.31 ± 0.02 B	***	*
		FtA	1.39 ± 0.26 c	1.46 ± 0.40 c	4.11 ± 0.24 b	5.55 ± 0.81 a	1.38 ± 0.05 C	2.98 ± 0.19 B	3.42 ± 0.13 A	3.28 ± 0.12 A	*	***
		FA	0.33 ± 0.02 b	0.49 ± 0.01 a	0.52 ± 0.02 a	nd	nd	nd	nd	nd	ns	ns
		GA	27.26 ± 0.62 b	29.37 ± 1.16 a	24.04 ± 0.58 c	18.69 ± 0.13 d	22.45 ± 0.66 A	22.58 ± 1.50 A	20.17 ± 0.48 B	21.44 ±1.30 AB	***	***
Stilbens		R	1.12 ± 0.11 b	1.09 ± 0.06 b	2.19 ± 0.13 a	2.48 ± 0.27 a	0.83 ± 0.03 C	1.75 ± 0.07 A	1.54 ± 0.05 B	1.40 ± 0.13 B	***	***
		RG	1.82 ± 0.36 d	2.26 ± 0.20 c	2.94 ± 0.09 b	4.01 ± 0.15 a	0.81 ± 0.02 D	1.15 ± 0.06 C	2.29 ± 0.03 B	2.48 ± 0.13 A	***	*

Two-way ANOVA shows the mean separation of the phenolic acids and stilbene components of Plavac Mali wines produced from berries harvested at four different dates (H1, H2, H3, and H4) at two locations (L) (Split and Zadar) and interactive effect (HD × L) of these factors (ns—not significant; * *p* ≤ 0.05; *** *p* ≤ 0.001). One-way ANOVA was done separately for each vineyard location. Mean values ± standard deviation (*n* = 3) within the same line, followed by different lowercase and uppercase Latin letters, indicate significant differences according to Fisher’s LSD test at *p* ≤ 0.05 among different harvest dates for Split and Zadar, respectively. CfT—caftaric, CA—caffeic acid, CtA—coutaric acid, CmA—coumaric acid, FtA—fertaric acid, FA—ferulic acid, nd – not detected, GA—galic acid, R—resveratrol, RG—resveratrol-glucoside. Values are presented in units of mg L^−1^.

## Data Availability

The data presented in this study are available on request from the corresponding author.

## Supplementary Materials

The following supporting information can be downloaded at: https://www.mdpi.com/article/10.3390/foods13172695/s1, Table S1: Evaluation of standard physicochemical parameters.
