# Supplementary material for: Positive Impact of Late Harvest Date on Polyphenolic Composition of Plavac Mali (Vitis vinifera L.) Wine Depends on Location
Source: Foods. 2024 Aug 26;13(17):2695. doi: 10.3390/foods13172695 (PMC11394399; doi:10.3390/foods13172695)
Supplement: Supplementary file 1 [file foods-13-02695-s001.zip › foods-3156053-supplementary.pdf]

Table S 1 Two-way and one-way ANOVA analysis of physicochemical parameters in the Plavac Mali wines of four different harvest dates from two locations, Split and Zadar, and the interactive effect of both factors.

Physicochemical parameter	**Split**				**Zadar**					
	**H1**	**H2**	**H3**	**H4**	**H1**	**H2**	**H3**	**H4**	**L**	**HD x L**
Ethanol (% v/v)	9.90 ± 0.10 d	11.99 ± 0.10 c	12.83 ± 0.00 b	14.37± 0.60 a	8.69 ± 0.00 D	10.85 ± 0.00 C	12.22 ± 0.00 A	11.59 ± 0.10 B	***	***
TDE (g L^{-1})	26.50 ± 0.36 b	26.73 ± 0.12 b	27.40 ± 0.00 b	42.43 ± 8.35 a	24.00 ± 0.00 C	23.40 ± 0.17 D	25.43 ± 0.12 B	26.50 ± 0.36 A	**	*
RS (g L^{-1})	1.50 ± 0.12 b	1.97 ± 0.12 b	2.02 ± 0.06 b	14.07 ± 8.72 a	2.10 ± 0.08 AB	2.04 ± 0.82 AB	2.71 ± 0.12 A	1.88 ± 0.09 B	*	*
Ash	2.96 ± 0.02 c	3.18 ± 0.04 b	3.02 ± 0.02 c	3.45 ± 0.06 a	2.30 ± 0.01 A	2.16 ± 0.03 C	2.22 ± 0 .03 B	2.33 ± 0.03 A	***	***
pH	3.45 ± 0.01 d	3.65 ± 0.00 b	3.59 ± 0.02 c	3.71 ± 0.02 a	3.26 ± 0.00 D	3.34 ± 0.01 C	3.39 ± 0.01 A	3.38 ± 0.01 B	***	***
TA (g L^{-1})	6.43 ± 0.06 a	5.63 ± 0.15 c	5.70 ± 0.00 c	5.90 ± 0 b	7.70 ± 0.00 A	6.47 ± 0.06 BC	6.43 ± 0.06 C	6.53 ± 0.06 B	***	***
VA (g L^{-1})	0.30 ± 0.01 c	0.34 ± 0.02 b	0.35 ± 0.01 b	0.47 ± 0.02 a	0.29 ± 0.01 B	0.25 ± 0.02 C	0.25 ± 0.00 C	0.44 ± 0.02 A	***	***

Two way ANOVA showing mean separation of the standard components of Plavac Mali wines produced from berries harvested at four different harvest dates (H1, H2, H3 and H4) on two locations (L) (Split and Zadar) and interactive effect (HD x L) of these factors (ns – not significant; * $p \leq 0.05$; ** $p \leq 0.01$; *** $p \leq 0.001$). One way ANOVA is done for each vineyard location separately. Mean values ± standard deviation (n = 3) within the same line followed by different lowercase and uppercase Latin letters (a, b, c, d) indicate significant differences according to Fisher's LSD test at $p \leq 0.05$ among different harvest dates for Split and Zadar, respectively. TDE – total dry extract; RS – reducing sugars; TA - total acidity expressed as (g L^{-1}) tartaric acid equivalents¸ VA - volatile acidity expressed as (g L^{-1}) acetic acid equivalents
